# Performance analysis and prediction of asphalt pavement containing municipal solid waste incineration bottom ash aggregate based on MEPDG

**DOI:** 10.1038/s41598-025-91686-6

**Published:** 2025-03-04

**Authors:** Yu Sun, Luo Ji, Jian Liao, Fulong Zhang, Yan Gao, Chongwei Huang

**Affiliations:** 1 Department of Transportation Engineering, University of Shanghai for Science and Technology, Shanghai, China; 2 Shanghai Pudong Road and Bridge Group Co, Ltd, Shanghai, China; 3 Shanghai Lucheng Intelligent Technology Development Co., Ltd, Shanghai, China

**Keywords:** Road engineering, Solid waste resource utilization, Municipal solid waste incineration (MSWI) bottom ash(BA), Mechanistic-empirical pavement design guide (MEPDG), Civil engineering, Environmental impact

## Abstract

Municipal solid waste incinerator (MSWI) bottom ash (BA) is the main product of municipal solid waste after being burned. MSWI bottom ash aggregate (BAA) which is made from processed BA can be used in road engineering due to its strength and gradation. And it can be provide more choices for road engineering aggregates and relieve the demands for natural aggregates in road engineering construction. In order to verify the difference between BA asphalt pavement and ordinary asphalt pavement in engineering practice, the Mechanistic-Empirical Pavement Design Guide (MEPDG) was used to analyze and predict the road performance of BA asphalt pavement, such as rutting, fatigue cracking, temperature cracking, and pavement smoothness. The results showed that the incorporation of MSWI BA had a great impact on the rutting depth of the pavement, but was less affected by temperature changes and had little effect on the fatigue cracking and smoothness of the pavement. Overall, MSWI BA did not degrade the long-term performance of asphalt pavements and is suitable for replacing part of the natural aggregates for road construction.

## Introduction

**Fig. 1:**
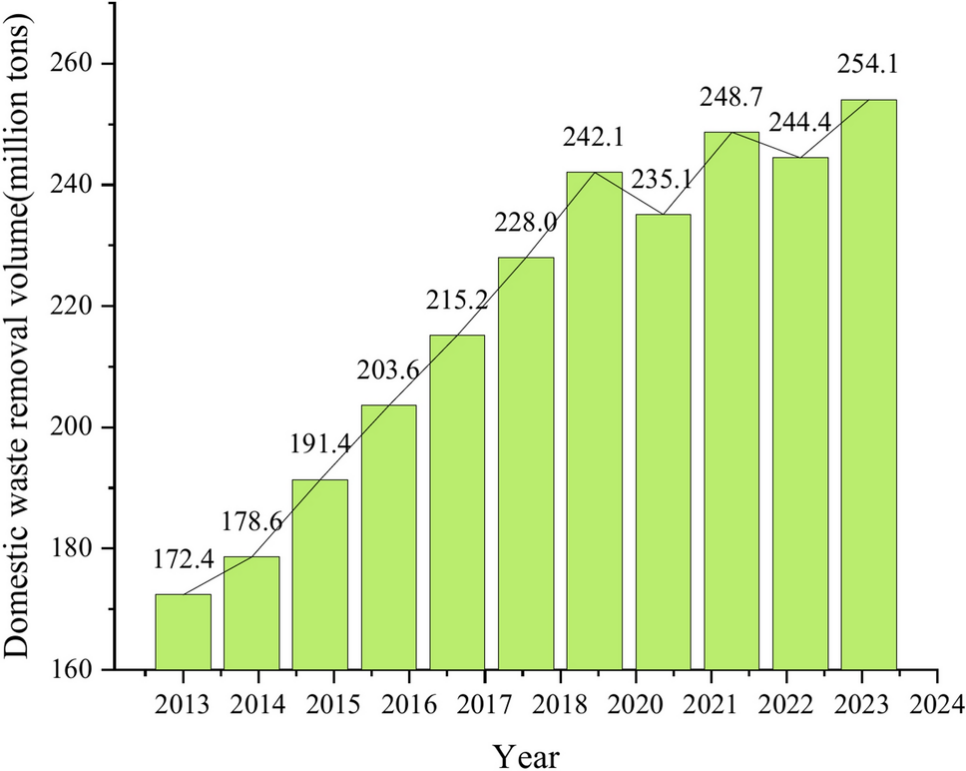
The annual waste removal volume (2013–2023).

The world is currently facing severe environmental challenges, with the development of society and population growth^1^, the production of solid waste is increasing, a and its generation is much larger than its disposal. It is estimated that by 2050, the global waste production will reach 3.4 billion tons^2^. Relevant data show that the annual waste removal volume is increasing, as shown in Fig. 1. MSWI BA is the main solid product of municipal solid waste after incineration, and its production is about 20% of the amount of waste in the factory^3^. In 2023, the amount of harmless treatment of municipal solid waste in China will be 259 million tons, and the annual output of BA will be about 52 million tons. In the process of MSWI BA formation, it experiences high-temperature roasting and is finally cooled and condensed. The particles are compact, and the mud content is small. When used as pavement material, the BA can show its high strength and high performance. It can not only alleviate the shortage of road construction materials but also solve the problem of waste slag treatment and land occupation and simultaneously reduce the emission of pollutants^4,5^.

MSWI BA is produced in huge quantities and at low prices. According to the market price in 2023, the unit price of one ton of MSWI BAA is 6.5 RMB, and the price of natural aggregate is 30 times more expensive, using MSWI BAA can save the cost of raw material purchase. At present, the resource utilization of BA has been explored in many fields. Some European countries have used more than 55% of BA to replace embankment filler and base aggregate in road engineering^6,7^. In addition, the BA can also be used to make ceramics and bricks, adsorb organic pollutants in rainwater and surface runoff as adsorbents, apply to landfill cover materials, prepare hydrogen, etc., and even improve soil as an agricultural fertilizer^8–10^. And MSWI BA asphalt pavement has also been paved and put into use in Nanjing, Shanghai and other places.

Although MSWI BAA is close to natural aggregate, on the whole, it also shows some differences compared with natural aggregate. Many scholars have investigated the feasibility of replacing natural aggregates with MSWI BAA to meet the problem of shortage of natural aggregates in road engineering. However, most of the current research work is are exploring the road performance of MSWI BA asphalt mixtures through laboratory tests^11–17^. There were also road engineering-related companies that have paved experimental roads to observe the long-term performance of asphalt pavements^18–20^, which is more reliable but time-consuming and costly to actually observe on the road. Because MSWI BAA is characterized by acidic aggregate and complex chemical composition, it will affect the long-term performance of MSWI BA asphalt pavement. The simulation and prediction of the performance of MSWI BA asphalt pavement in advance can provide data support and basis for subsequent construction, and reduce the construction cost. Joumblat et al.^21^ simulated the long-term rutting performance of MSWI FA, but there were few studies on the overall prediction and analysis of the road performance of MSWI BA asphalt pavement, so the research in this paper is meaningful.

In this paper, through the combination design of the pavement structure, the prediction models were used to analyze and predict the performance of asphalt pavement containing MSWI BAA, including rutting, fatigue crack, temperature crack, and pavement smoothness based on the Mechanistic-Empirical Pavement Design Guide (MEPDG). Finally, the results were compared with those of ordinary asphalt pavements.

## Municipal solid waste incinerator bottom ash aggregate (MSWI BAA)

### The scope of MSWI BA

Under the background of rapid global economic and social development, the total amount of municipal solid waste is increasing at a rate of 8%−10% per year. The incineration method has become one of the most important ways to deal with municipal solid waste by virtue of its significant advantages of reduction and resource utilization. And the studies have shown that the quality of municipal solid waste after incineration can be reduced by 65%−80%, and the volume can be reduced by 85%−90%^22^. MSWI BA accounts for about 80% of the production of municipal solid waste incineration residue. According to relevant data, the global annual MSWI BA production is about 250 million tons, of which China ‘s annual MSWI BA production is 150 million tons, while the recycling rate of MSWI BA is only 20%, so the recycling market potential of MSWI BA is huge.

### The mhemical composition and basic properties of MSWI BAA

**Fig. 2:**
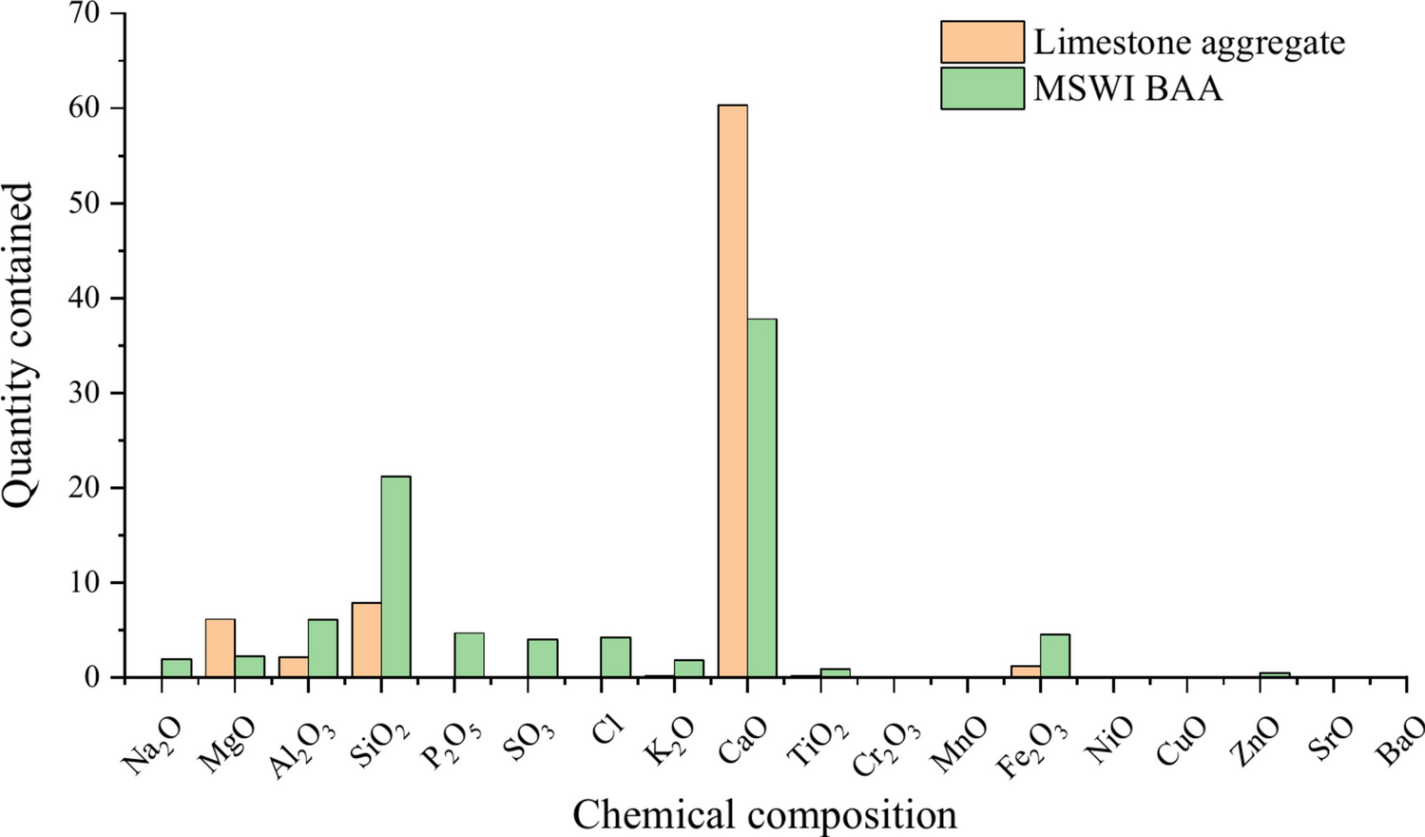
Chemical composition of limestone aggregate and MSWI BAA.

MSWI BAA is mainly a complex mixture composed of glass, ceramics, masonry, slag and other substances. Among the main components of MSWI BAA, the highest content is Ca element, followed by Si element. In addition, it also contains a small amount of Na, Mg, Al, P, S, Fe and other elements^23^. The composition is much more complex than natural aggregate. The chemical composition of limestone aggregate and MSWI BAA is shown in Fig. 2.

According to the existing laboratory test results^24^, it can be seen that the particle size distribution of the MSWI BAA at all levels is uniform, with a certain proportion, and the grading curve is smooth and continuous, which belongs to the continuous grading type of aggregate. The basic properties test results of the MSWI BAA are shown in Table 1.

**Table 1:** Basic properties of MSWI BAA.

Particle size/mm	Apparent relative density	Absorption rate	Crushing value	Abrasion value	pH
0–2.36	2.667	-	32.2	-	-
2.36–4.75	2.410	8.18	38.7	37.9	12.4

### The mix proportion design of MSWI BA asphalt mixture

Referring to the previous research results on the influence of MSWI BA particle size range and dosage in BA asphalt mixture, it is recommended that the BAA particle size range is 0–2.36 mm and the dosage is 10% in SMA asphalt mixture, and the BAA particle size range is 0–9.5 mm and the dosage is 20% in fine-grained AC asphalt mixture^25–28^. In this paper, the optimal recommended particle size and content are selected to design the composition of MSWI BA asphalt mixture. The synthetic gradation of MSWI BA asphalt mixture is shown in Table 2. Then, the effective asphalt content of the corresponding mixture could be calculated, as given in Table 3.

**Table 2:** Synthetic grading of asphalt mixtures.

Mixture type	MSWI BAA dosage (%)	Mass percent (%) through the following sieve holes (mm)											
		26.5	19	16	13.2	9.5	4.75	2.36	1.18	0.6	0.3	0.15	0.07
SMA-13	0	100	100	100	94.6	62.5	28.0	21.8	18.2	16.3	14.4	13.2	11.9
	10	100	100	100	94.6	62.5	26.1	16.5	14.4	12.6	11.6	10.8	10.3
AC-16	0	100	100	99.6	92.0	65.2	48.8	34.9	22.6	16.4	11.1	9.2	9.2
	20	100	100	99.6	92.0	65.5	48.1	31.6	21.7	16.0	11.8	9.3	8.6
AC-20	0	100	98.2	87.2	70.6	59.6	42.3	26.8	18.2	15.3	8.9	6.1	2.4
	20	100	98.1	86.8	69.9	59.6	42.2	26.3	17.7	13.4	7.2	4.3	1.7
Base	—	100	95.8	88.4	80.5	69.7	27.2	23.4	—	12.5	—	—	3.15

**Table 3:** The effective asphalt content of asphalt mixture.

Material	MSWI BAA dosage(%)	Effective asphalt content (%)
SMA-13	0	5.27
	10	5.80
AC-16	0	3.14
	20	4.90
AC-20	0	3.08
	20	4.48

### The road performance of MSWI BA asphalt mixture

According to the results of the previous laboratory test^28^ and the design results of the mixture ratio of Sect. “The mix proportion design of MSWI BA asphalt mixture”, the road performance of the MSWI BA asphalt mixture is evaluated by rutting test, low temperature bending test and freeze–thaw splitting test, and compared with the original asphalt mixture to clarify the effect of MSWI BAA on the road performance of asphalt mixture. The road performance test results are shown in Table 4.

**Table 4:** Road performance of MSWI BA asphalt mixture.

Material	MSWI BAA dosage (%)	Dynamic stability ( times·mm-1 )	Maximum bending strain (με)	TSR (%)
SMA-13	0	6769	6225	83.1
	10	6501	4639	96.7
AC-16	0	1508	2671	85.2
	20	2233	2853	89.5
AC-20	0	1916	2428	81.7
	20	1208	2522	72.0

The incorporation of BA will improve the high temperature stability and water stability of asphalt mixture to a certain extent, but it will not have a great impact on the low temperature crack resistance of asphalt mixture.

## Modeling

### Scope of application of MEPDG

In March 1996, the United States AASHTO Joint Task Force on Pavements, NCHRP and FHWA held a conference in California conducted a comprehensive discussion on pavement design, aiming to find a unified pavement design standard. In March 2004, NCHRP 01-37A research group published the Mechanistic-Empirical Pavement Design Guide (MEPDG). In July of the same year, MEPDG design software began to trial. Since then, NCHRP has continued to improve MEPDG and its software. In April 2011, the latest version of the design program DARWin-ME was published. At present, MEPDG has been adopted and promoted in more than 40 states in the United States and has attracted worldwide attention. MEPDG is one of the most commonly used empirical design methods of pavements machinery^29^based on the principle of mechanics and experience, and it provides a unified basis for the design of flexible pavement, rigid pavement and composite pavement, and adopts common design parameters of traffic, subgrade, environment and reliability^30^. It can not only predict a variety of pavement performance, but also establish a link between materials, pavement structure design, construction, climate, traffic and pavement management system. Kong et al.^31^optimized the MEPDG rutting model by introducing a growth coefficient for the NLR and the research results could provide guidance for predicting the rutting depth of asphalt pavement throughout the life cycle. The MEPDG combines the advantages of mechanical method and empirical method, and has been corrected by a large number of data. The disadvantage of MEPDG is that it is necessary to input clear road climate information and traffic volume information when using software to simulate road performance, but in general, the pavement performance prediction model proposed by MEPDG is more comprehensive, and the factors considered in the calculation process are more comprehensive and objective. It can reflect the actual situation of the pavement more accurately^32–34^.

### Prediction model

In this paper, the rutting prediction model, fatigue crack prediction model, temperature crack prediction model and pavement roughness prediction model in MEPDG are selected as the representative evaluation index models of asphalt pavement performance for analysis and prediction.

#### Rutting prediction model

Rutting is a hollowing of the wheel path surface due to inelastic or plastic deformation of any or all pavement layers and subgrades. With the increase in road traffic volume, vehicle axle load overloading, and channelization, the rutting disease of asphalt pavement is becoming more and more serious. This, in turn, induces other diseases, greatly reducing the service life of asphalt pavement and affecting the serviceability of asphalt pavement^35^. The total rutting of MEPDG flexible pavement is the sum of all layers of pavement rutting, including pavement surface, base, and cushion. MEPDG assumes that there is no rutting in the consolidated layer formed by the material used to reinforce each layer in the pavement. The MEPDG prediction model of asphalt layer rutting is as Eq. (1):

$$\Delta_{p(HMA)} = \varepsilon_{p(HMA)} \cdot \varepsilon_{r(HMA)}$$

1 $$= \beta_{r1} k_{\tau } \varepsilon_{r(HMA)} \cdot 10^{ - 3.35412} T^{{1.5606\beta_{r2} }} N^{{ - 0.4791\beta_{r3} }} h_{HMA}$$

where $\Delta_{{p\left( {HMA} \right)}}$ is accumulated permanent deformation of the asphalt layer or sublayer, $\varepsilon_{{p\left( {HMA} \right)}}$ is the accumulative plastic axial strain of the asphalt layer or sublayer, $\varepsilon_{{r\left( {HMA} \right)}}$ is the central elastic strain of road surface, $k_{\tau }$ is the deep confining pressure coefficient, and $N$ is load times. $\beta_{r1}$, $\beta_{r2}$ and $\beta_{r3}$ represent the calibration coefficient.

#### Fatigue crack prediction model

Cyclic loads, such as vehicle loads, seismic loads, and cyclically varying water pressures, are applied to the pavement. The pavement will produce fatigue cracks under these cyclic loads, resulting in safety hazards in the normal use process. The prediction of fatigue crack by MEPDG is done in two steps. The first step is to calculate the fatigue damage index of the asphalt layer. The second step determines the quantitative relationship between fatigue damage index and fatigue crack, and calibrates the relevant parameters. The fatigue damage index is based on the Miner law and expressed by Eq. (2):

2 $$DI = \sum\limits_{i = 1}^{T} {\frac{{n_{i} }}{{N_{i} }}}$$

where $DI$ is fatigue damage index, $T$ is the period of load, $n_{i}$ is the number of traffic loads in cycle *i,* and $N_{i}$ is the number of allowed traffic loads in cycle *i*.

The bottom-up crack can be calculated by Eq. (3):

3 $$FC_{bottom} = \left( \frac{1}{60} \right)\left( {\frac{{C_{4} }}{{1 + e^{{\left( {C_{1} C_{1}^{*} + C_{2} C_{2}^{*} \log \left( {DI_{bottom} } \right)} \right)}} }}} \right)$$

where $FC_{bottom}$ is the bottom-up crack, and $DI_{bottom}$ is the bottom-up fatigue damage index.

$$C_{4} = 6000$$; $$C_{1} = 1$$, $$C_{1}^{*} = - 2C_{2}$$; $$C_{2} = 1.0$$, $$C_{2}^{*} = - 2.40876 - 39.748 \times \left( {1 + h_{ac} } \right)^{ - 2.856}$$

The up-down crack can be calculated by Eq. (4):

4 $$FC_{top} = 10.56\left( {\frac{{C_{4} }}{{1 + e^{{\left( {C_{1} - C_{2} \log \left( {DI_{top} } \right)} \right)}} }}} \right)$$

where $FC_{top}$ is the up-down crack, $DI_{top}$ is the up-down fatigue damage index, and $C_{1} = 7.0$, $C_{2} = 3.5$, $C_{4} = 1000$.

#### Temperature crack prediction model

Low-temperature cracking is the main failure mode of asphalt pavements in areas with cold and large temperature differences. The generation of temperature cracks affects the integrity, continuity, and aesthetics of the pavement, which will lead to the infiltration of water into the pavement structure through cracks, thereby weakening the adhesion between asphalt and aggregate, easily inducing other damages, and seriously shortening the service life of asphalt pavement^36^. MEPDG predicts asphalt concrete temperature cracks as shown in Eq. (5):

5 $$TC = \beta_{t1} N\left[ {\frac{1}{{\sigma_{d} }}\log \left( {\frac{{C_{d} }}{{h_{HMA} }}} \right)} \right]$$

where $TC$ is the temperature crack observed, $\beta_{t1}$ is the regression coefficient, $N\left( z \right)$ is the standard normal distribution at $z$, $\sigma_{d}$ is the standard deviation of logarithm of crack depth, and $C_{d}$ is the crack depth changes in a freezing cycle.

#### Pavement smoothness prediction model

Pavement smoothness is the deviation value of longitudinal bump on the pavement. With the development of road traffic, people reach their destination and also pursue the comfort and safety of driving. Therefore, pavement smoothness is one of the important inspection indexes that cannot be ignored in highway construction. IRI(International Roughness Index) estimates are incremental throughout the design period^37^. MEPDG predicts the smoothness IRI of flexible pavement by empirical Eq. (6):

6 $$IRI = IRI_{0} + 0.015\left( {SF} \right) + 0.400\left( {FC_{Total} } \right) + 0.0080\left( {TC} \right) + 40.0\left( {RD} \right)$$

where $IRI_{0}$ is the initial IRI after construction, $SF$ is the location factor, $FC_{Total}$ is the fatigue crack area, $RD$ is the average rut depth, and $TC$ is the transverse crack length (including existing asphalt pavement transverse reflection crack).

### Model parameter

#### Traffic data

MEPDG pavement design software needs to input the traffic volume of the designed road to proceed with calculations^38^. According to the traffic grade standard of asphalt pavement structure in ‘Shanghai urban road and highway design guidance’ and Code for Pavement Design of Urban Road (CJJ 169–2012), the intermediate values of cumulative axle times of the main and secondary trunk roads were selected as the design traffic volume, and the traffic load data were input as presented in Table 5 and Table 6.

**Table 5:** Asphalt pavement design specification design.

Road type	Cumulative equivalent number of axles N_c_ (ten thousand times / lane)	Resilient modulus of road soil foundation (MPa)
Main trunk road	1200–2500	≥ 30
Secondary trunk road	400–1200	≥ 25

**Table 6:** Traffic load on arterial and secondary arterial roads.

Road parameters	Main trunk road	Secondary trunk road
Initial bi-annual average daily traffic (pcu/d)	9930	3310
Number of lanes in design direction	2	2
Design direction truck proportion (%)	50	50
Design lane freight car ratio (%)	60	60
Driving speed (mph)	60	60
Traffic growth (%)	4	4

#### Climatic data

**Fig. 3:**
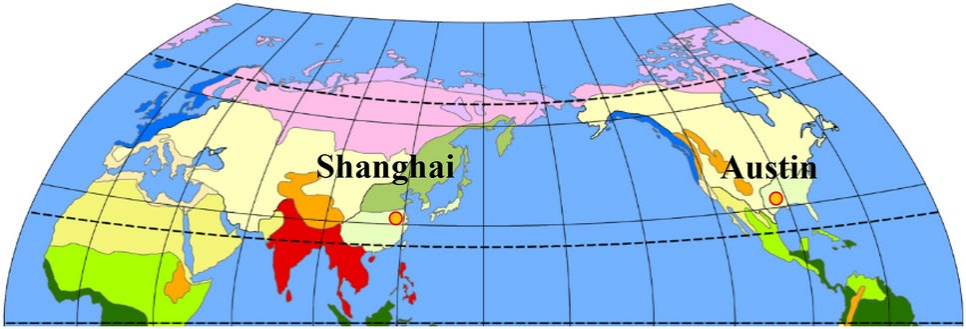
Geographical location and climate type.

**Fig. 4:**
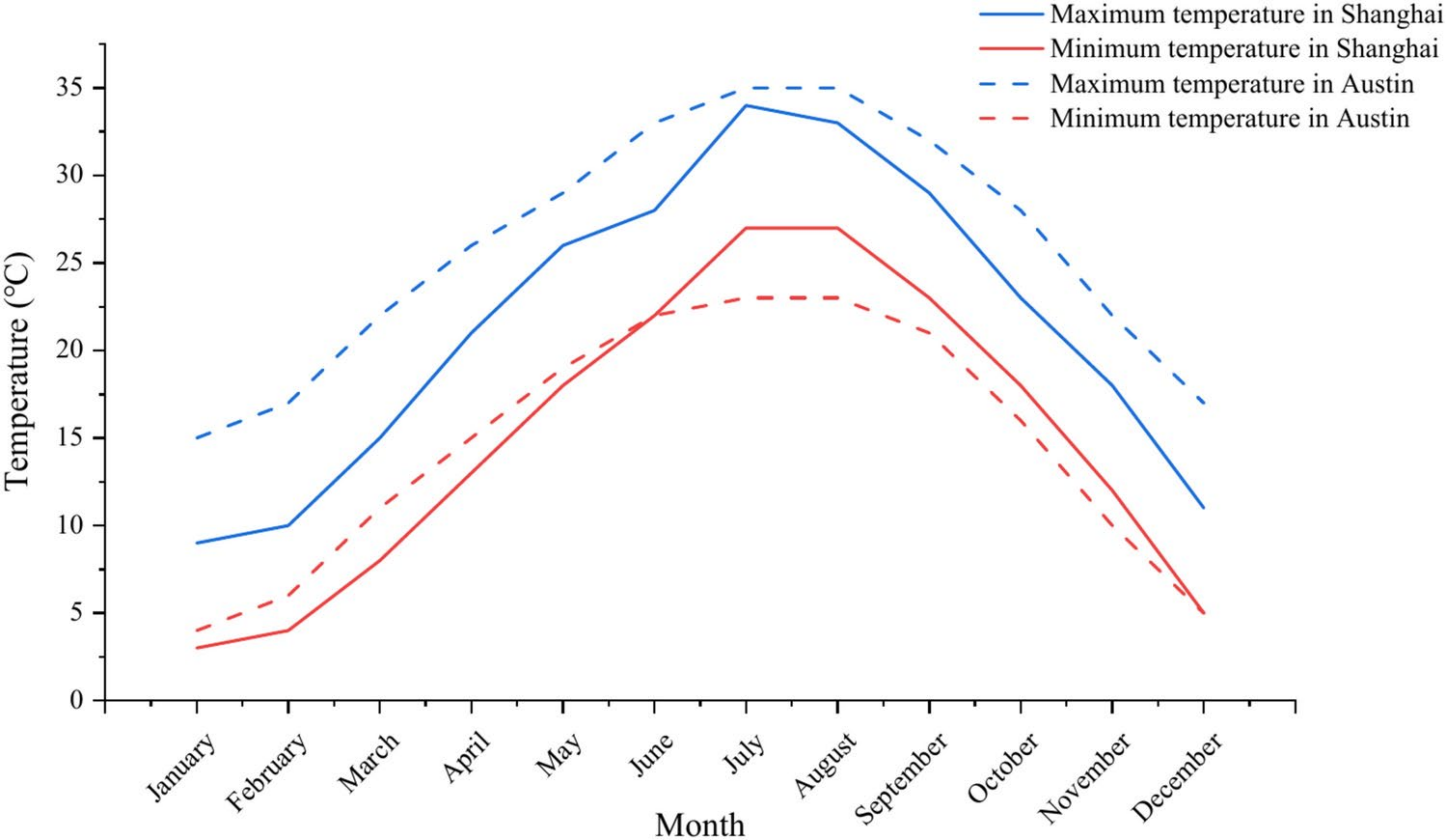
Monthly average maximum and minimum temperatures throughout the year.

MEPDG pavement design software needs to input climate information such as temperature and rainfall over the years. MEPDG has the climate data of many cities in North America, which are the actual meteorological information obtained by long-term observation of each meteorological station, and can be used for selection. The asphalt pavement containing MSWI BAA has been paved in Shanghai, Nanjing, Tianjin and other places in China^39^. This paper aims to verify the road performance of Shanghai test road, but the MEPDG database only has the climate data of the United States. It was found that Austin and Shanghai are both near 30°N latitude and have the same subtropical monsoon climate with four distinct seasons, sufficient sunshine and abundant rainfall after research, as shown in Fig. 3. Therefore, the climate data of Austin is selected as a reference in the software in this paper. The monthly average maximum and minimum temperatures of the two cities are shown in Fig. 4. The climate data inputed in MEPDG is shown in Table 7.

**Table 7:** Input climate data.

Latitude (degrees. minutes)	Longitude (degrees. minutes)	Elevation (ft)	Depth of water table (ft)
			Annual average
30.11	−97.41	661	12

#### Pavement structure

Based on the previous research results^24^, the optimum mix ratio design of the asphalt pavement containing MSWI BAA has been determined, and the pavement structure design has been carried out accordingly. In order to simulate the performance of MSWI BA asphalt pavement for different grades of roads, two structural combinations of the main and secondary trunk roads were selected. The main trunk road from the bottom to top comprised cushion, base, lower layer, middle layer, and upper layer. When the asphalt surface layer consists of multiple layers, the maximum particle size of the aggregate should increase layer by layer from top to bottom and match the design layer thickness. Therefore, the thickness of each layer was calculated in conjunction with the nominal maximum particle size when setting up the roadway surface structure. In this regard, SMA-13, AC-16, and AC-20, with thicknesses of 4, 6, and 8 cm, were selected as the upper, middle, and lower layers of asphalt concrete materials, respectively. The secondary trunk road from the bottom to top consisted of a cushion, base, lower layer, and upper layer; SMA-13 and AC-20 with thicknesses of 4 and 8 cm were selected as the upper and lower layers of asphalt concrete materials, respectively. Two kinds of pavement structure combinations set the same base (35 cm), and the cushion was directly set to the bottom without setting thickness. The specific structural combinations and Poisson’s ratio of each layer are shown in Table 8.

**Table 8:** The pavement structure of arterial and secondary arterial roads.

Structure level	Main trunk road		Secondary trunk road		Poisson’s ratio
	Material	Thickness	Material	Thickness	
The upper layer	SMA-13	4 cm	SMA-13	4 cm	0.25
The middle layer	AC-16	6 cm	—	—	0.25
The lower layer	AC-20	8 cm	AC-20	8 cm	0.25
Base	SW-SM	35 cm	SW-SM	35 cm	0.25
Cushion	Gravel	—	Gravel	—	0.3

#### Design parameters of each layer of material

**Fig. 5:**
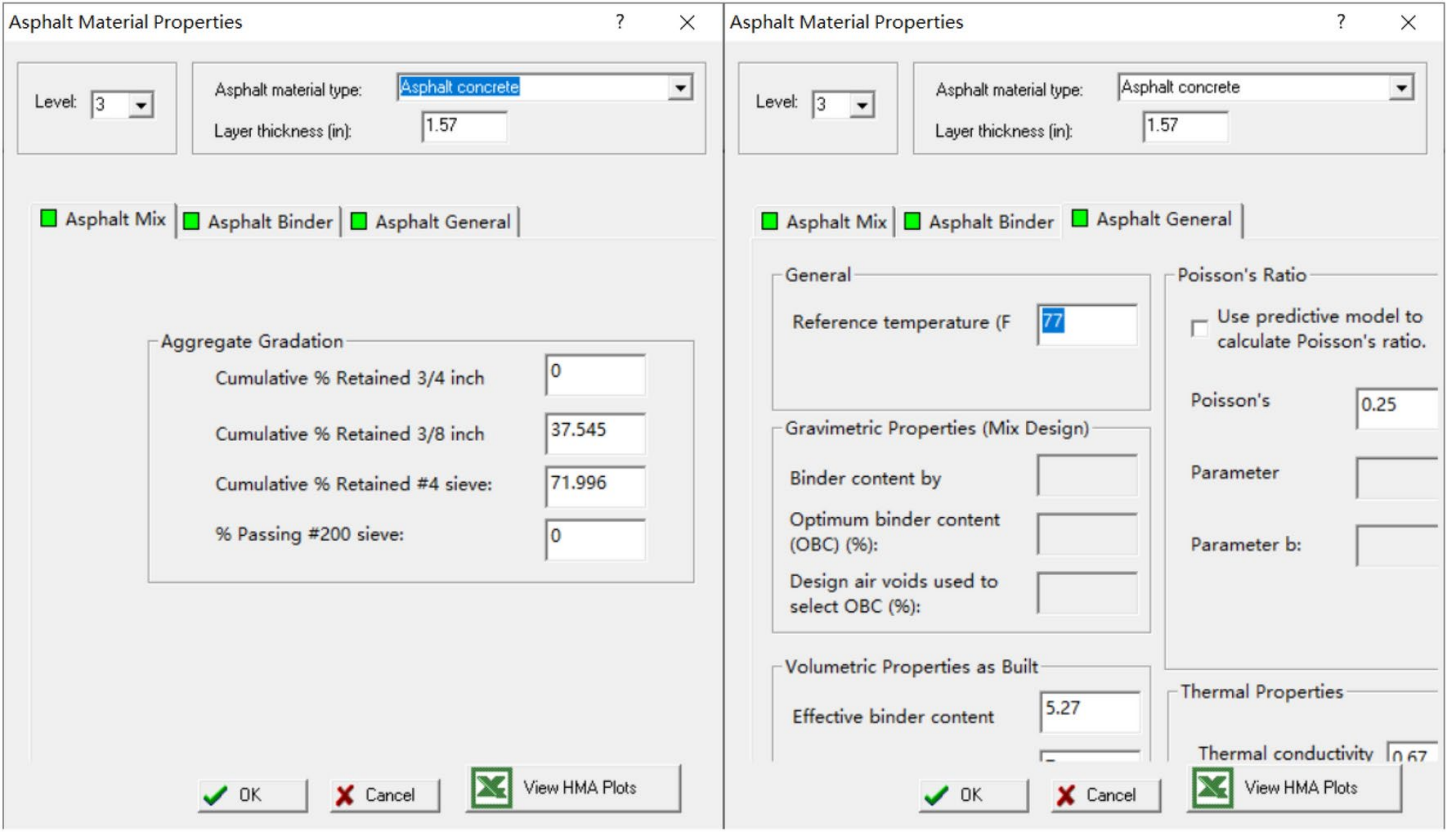
The operation interface of the input material parameters.

The MEPDG also requires the input of the design parameters of each layer of material, including the parameters required for the calculation of road response such as material modulus, the parameters required for performance conversion equations such as tensile strength, and the material parameters required for environmental models such as temperature conductivity. Figure 5 is the operation interface of the input material parameters.

Since the MEPDG software comes with the default values of various road material parameters, this paper adjusts some parameters according to the characteristics of MSWI BA.Table 9 is the difference between the material design parameters of the ordinary pavement and MSWI BA asphalt pavenment for main trunk road, and Table 10 is between the material design parameters of the ordinary pavement and MSWI BA asphalt pavenment for the secondary trunk road.

**Table 9:** Material design parameters for ordinary pavements and mswi ba asphalt pavenment on main trunk road.

Input parameter			Main trunk road	
			Ordinary pavement	MSWI BA asphalt pavenment
The upper layer	General Properties	Layer thickness (in)	1.57	1.57
		Reference temperature (F¡ã)	77	77
		Effective binder content (%)	5.27	5.8
	Asphalt Mix	Cumulative % Retained 3/4 inch sieve	0	0
		Cumulative % Retained 3/8 inch sieve	37.545	37.543
		Cumulative % Retained #4 sieve	71.996	73.857
		% Passing #200 sieve	0	0
	Thermal Cracking Properties	Average Tensile Strength at 14ºF	350	909.66
		Mixture VMA (%)	19.5	12.8
The middle layer	General Properties	Layer thickness (in)	2.36	2.36
		Effective binder content (%)	3.14	4.9
	Asphalt Mix	Cumulative % Retained 3/4 inch sieve	0	0
		Cumulative % Retained 3/8 inch sieve	34.763	34.451
		Cumulative % Retained #4 sieve	51.223	51.869
		% Passing #200 sieve	0	0
The lower layer	General Properties	Layer thickness (in)	3.15	3.15
		Effective binder content (%)	3.08	4.48
	Asphalt Mix	Cumulative % Retained 3/4 inch sieve	1.848	1.904
		Cumulative % Retained 3/8 inch sieve	40.445	40.44
		Cumulative % Retained #4 sieve	57.61	57.765
		% Passing #200 sieve	0	0

**Table 10:** Material design parameters for ordinary pavements and mswi ba asphalt pavenment on secondary trunk road.

Input parameter			Secondary trunk road	
			Ordinary pavement	MSWI BA asphalt pavenment
The upper layer	General Properties	Layer thickness (in)	1.57	
		Effective binder content (%)	5.27	5.8
	Asphalt Mix	Cumulative % Retained 3/4 inch sieve	0	0
		Cumulative % Retained 3/8 inch sieve	37.545	37.543
		Cumulative % Retained #4 sieve	71.996	73.857
		% Passing #200 sieve	0	0
	Thermal Cracking Properties	Average Tensile Strength at 14ºF	350	909.66
		Mixture VMA (%)	19.5	12.8
The lower layer	General Properties	Layer thickness (in)	3.15	3.15
		Effective binder content (%)	3.08	4.48
	Asphalt Mix	Cumulative % Retained 3/4 inch sieve	1.848	1.904
		Cumulative % Retained 3/8 inch sieve	40.445	40.44
		Cumulative % Retained #4 sieve	57.61	57.765
		% Passing #200 sieve	0	0

## Results and discussion

### Rutting

**Fig. 6:**
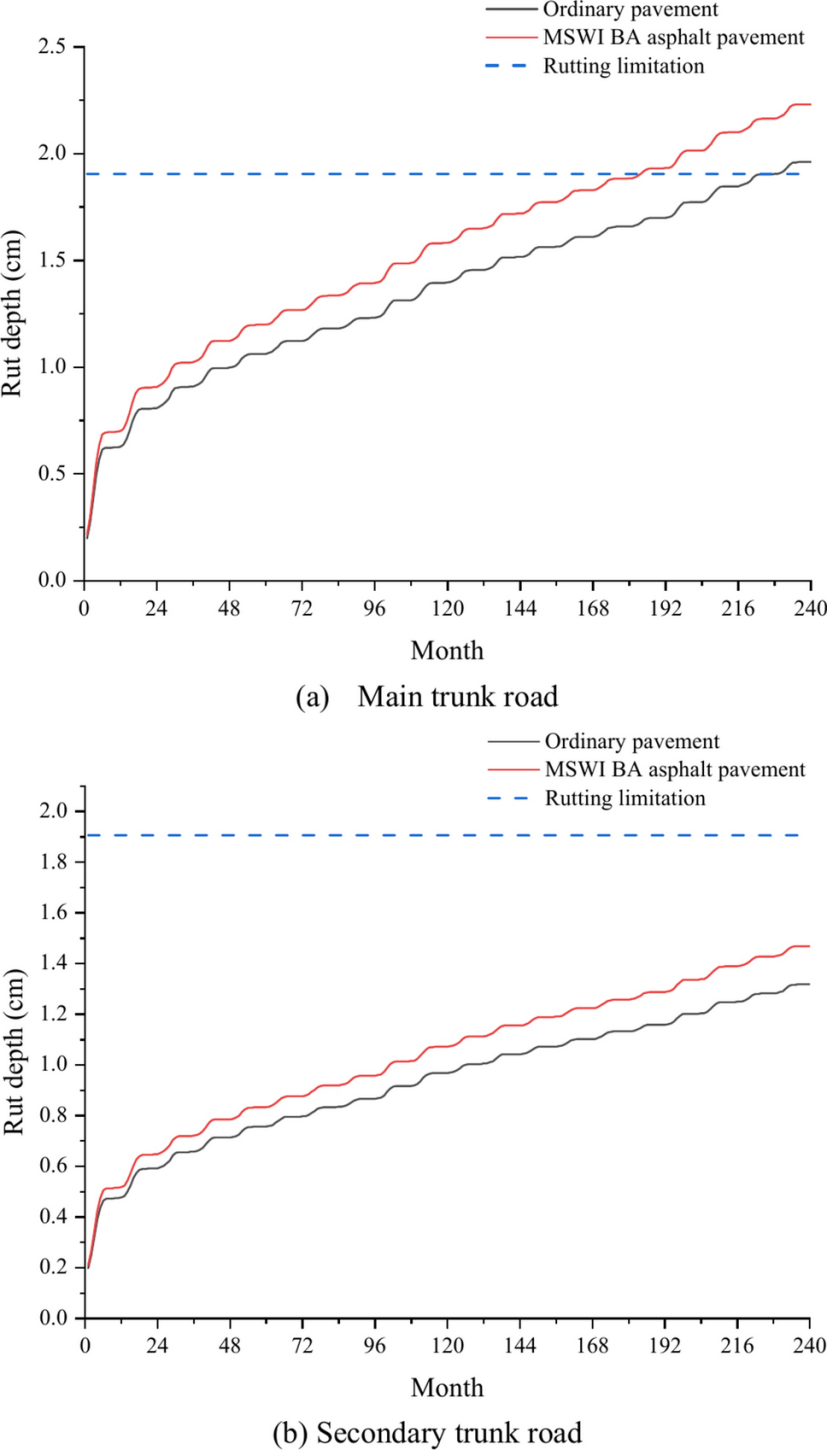
Prediction results of rutting.

As seen in Fig. 6, the rut depths of the four combinations increased with time in a fluctuating manner. The rut depth increased rapidly in the first eight months, and then the rut depth increased slowly. For the main trunk road, the rut depth of ordinary pavement exceeded the rutting limitation for the first time in the 228th month, and the rut depth was 1.96 cm in the 240th month. The rut depth of asphalt pavement containing MSWI BAA exceeded the rutting limitation for the first time in the 184th month, and the rut depth was 2.23 cm in the 240th month. For the secondary trunk road, the rut depth of the ordinary road did not exceed the rutting limitation within 240 months, and the rut depth in the 240th month was 1.32 cm. The rut depth of asphalt pavement containing MSWI BAA did not exceed the rutting limitation within 240 months, and the rut depth was 1.47 cm in the 240th month.

The rut depth of the main and secondary trunk roads of asphalt pavement containing MSWI BAA was slightly increased compared with the ordinary pavement. The maximum rut depth of the main trunk road increased from 1.96 to 2.23 cm, an increase of 13.3%, and the maximum rut depth of the secondary trunk road increased from 1.32 cm to 1.47 cm, an increase of 10.7%. The rut depth of the main trunk road increased significantly compared with the secondary trunk road with a small traffic volume, and the maximum rut wheel depth of the ordinary road increased from 1.32 cm to 1.96 cm, with an increase of 47.6%. The maximum rut wheel depth of asphalt pavement containing MSWI BAA increased from 1.47 to 2.23 cm, an increase of 50.9%.

**Fig. 7:**
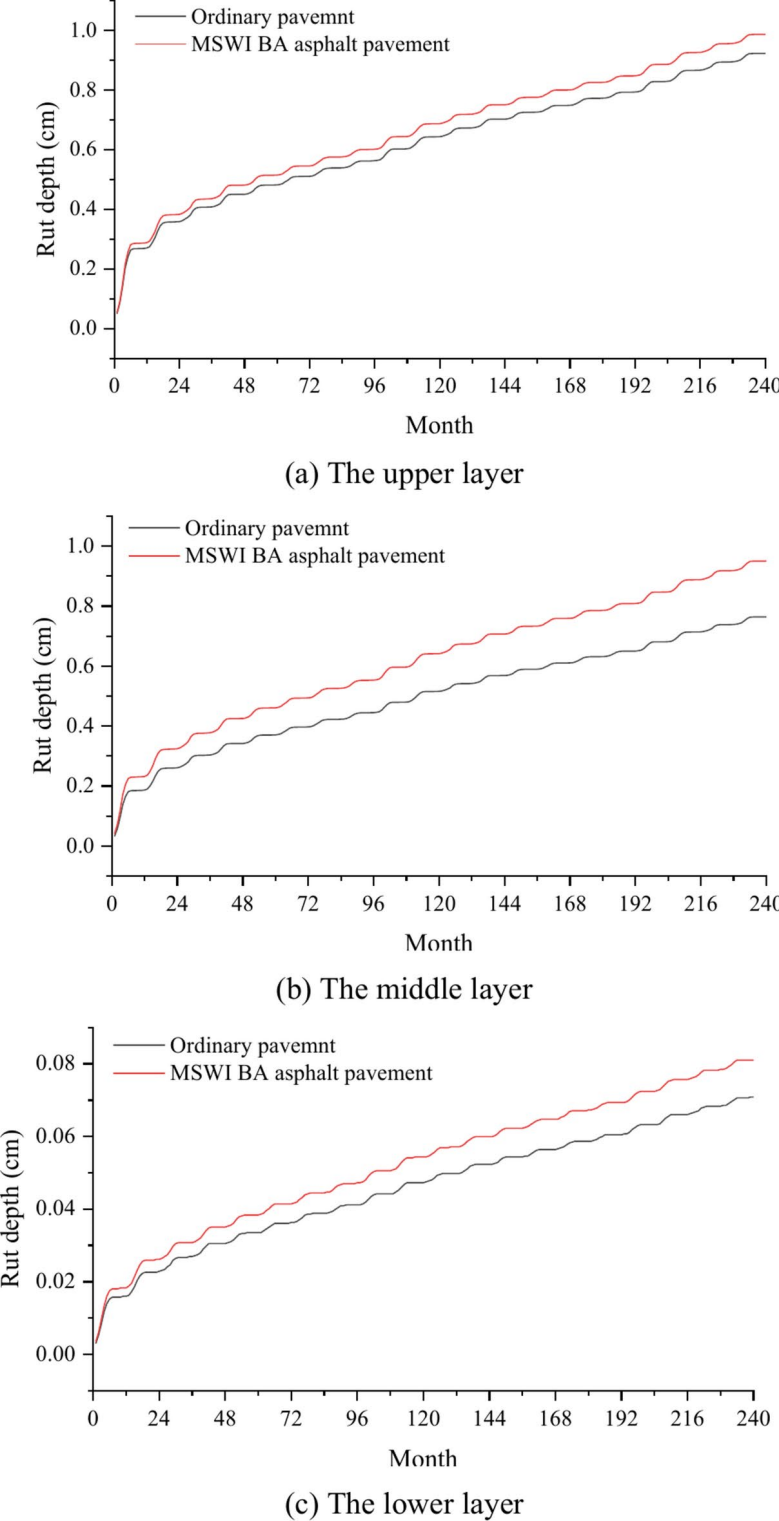
Prediction results of each layer’s rutting.

Figure 7 shows the curves of the rut depth of each surface layer as a function of time on two different main trunk roads: ordinary pavement and asphalt pavement containing MSWI BAA. As can be seen in Fig. 7, the rut depth of each surface layer gradually increased within the estimated 240 months. For the ordinary pavement, the rut depth growth rate of the lower layer was relatively stable. The rut depth was much smaller than thase of the middle and upper layers, and increased to 0.07 cm in the 240th month. The rut depth of the upper and middle layers increased greatly in the first eight months, and then the growth was stable. By the 240th month, the rut depth of the middle layer increased to 0.764 cm, and the rut depth of the upper layer increased to 0.923 cm.

The rut depth growth rate of the lower layer of the asphalt pavement containing MSWI BAA was also relatively stable compared with the ordinary pavement. The rut depth was much smaller than that of the middle and upper layers, which increased from 0.071 to 0.081 cm, incrementing by 0.01 cm, and the growth rate was 14.1%. In the first seven months, the rut depth of the middle layer increased rapidly, and then increased steadily. The rut depth was very close to the upper layer rut, from 0.764 to 0.95 cm, increased by 0.186 cm, and the growth rate was 24.3%. In the first seven months, the rut depth of the upper layer increased greatly, and then increased steadily. The rut depth increased from 0.932 to 0.987 cm, which increased by 0.055 cm, and the growth rate was 5.9%.

It can be seen that incorporating MSWI BA impacted the rutting depth of the pavement. This is because MSWI BA has more pores and large water absorption, which makes the asphalt content of MSWI BA asphalt mixture higher^28^, leading to a decrease in the rutting resistance of MSWI BAA. On the one hand, the influence of traffic volume on the rut depth was more prominent, because the load of the main trunk road was more times and the load was larger. On the other hand, because the material of the middle layer and the lower layer was of a suspended dense structure, there was no skeleton inlaid, so the anti-rutting performance was poor. In summary, after incorporating MSWI BA for the main trunk road, it exceeded the rutting limit advance for the first time in the 44th month. However, the addition of MSWI BA has little effct on the secondary trunk road, and the predicted rutting depth did not exceed the rutting limit within 240 months. Obviously, it can be seen that asphalt pavement containing MSWI BAA is more suitable for the secondary trunk roads with small traffic volume, and the increase of rut on the asphalt pavement containing MSWI BAA mainly came from the middle layer. This is consistent with the conclusion of Joumblat ‘s research^21^ that ‘*the use of MSWI-FA as a replacement of virgin fine aggregates negatively affects the resistance of AC mixtures to rutting*’.

### Fatigue crack

**Fig. 8:**
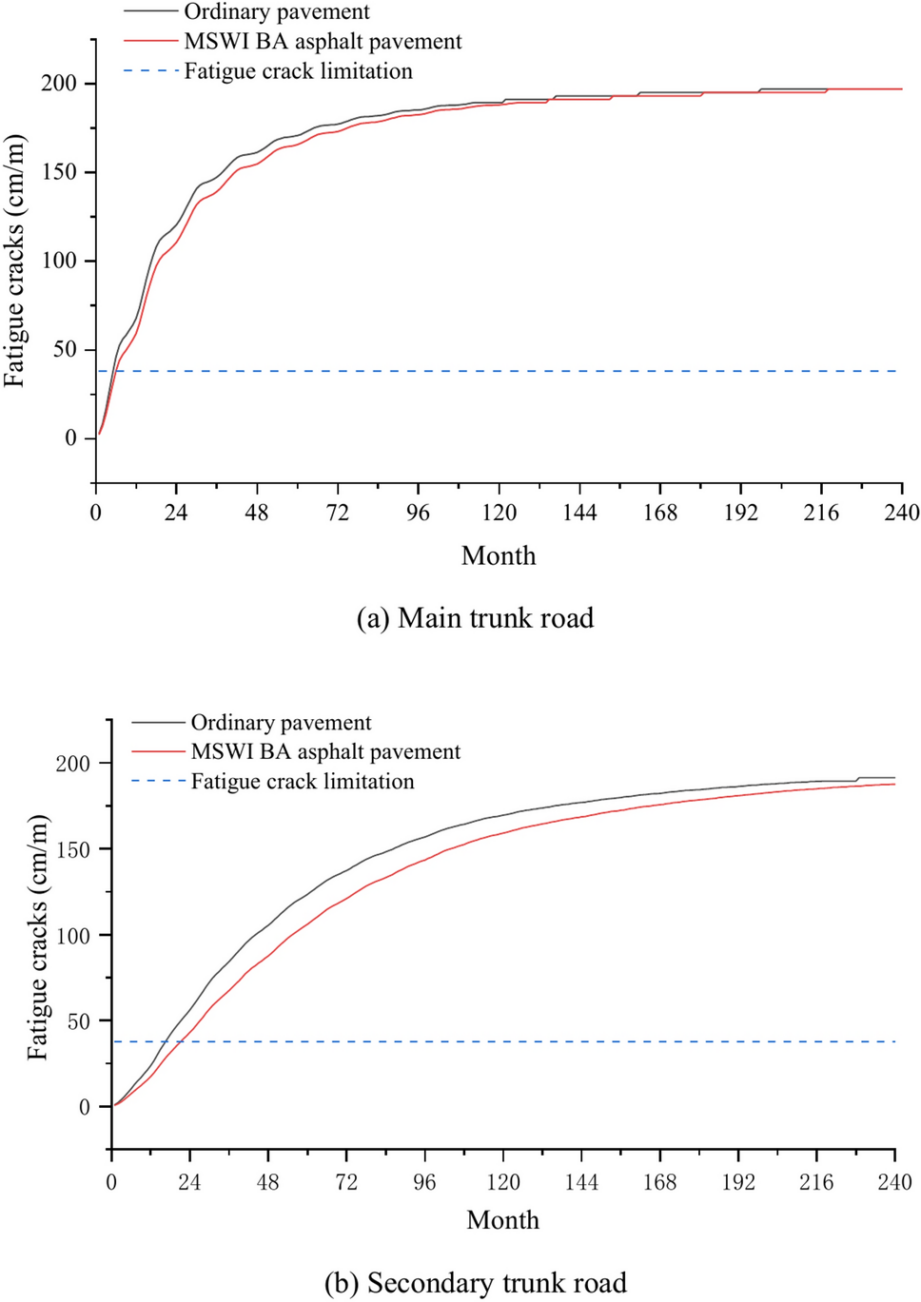
Prediction results of fatigue crack.

Figure 8 shows the fatigue cracks curves of ordinary pavement and asphalt pavement containing MSWI BAA for the main and secondary trunk roads with time. As can be seen, the fatigue cracks of the four combinations gradually increased with time. For the main trunk road, the common road and asphalt pavement containing MSWI BAA exceeded the fatigue crack limitation for the first time in the sixth month, and the fatigue cracks were 196.97 cm/m in the 240th month. For the secondary trunk road, the fatigue crack of the ordinary road exceeded the fatigue crack limitation for the first time in 17 months, and the fatigue crack was 191.29 cm/m in 240 months. The fatigue crack of the asphalt pavement containing MSWI BAA exceeded the fatigue crack limitation for the first time in 22 months, and the fatigue crack was 187.5 cm/m in 240 months.

Compared with the ordinary pavement, the fatigue cracks of the main trunk road of asphalt pavement containing MSWI BAA were not significantly different. In contrast, the fatigue cracks of the secondary trunk road were slightly reduced, and the fatigue cracks were reduced from 191.29 to 187.59 cm/m, with a decrease of 1.9%. In addition, the fatigue crack of the main trunk road slightly increased compared to the secondary trunk road. The fatigue crack of the ordinary road increased from 191.29 to 196.97 cm/m, with an increase of 3.0%, and that of the asphalt pavement containing MSWI BAA increased from 187.59 to 196.97 cm/m, with an increase of 5.0%. It can be seen that whether MSWI BA was mixed had no obvious effect on fatigue cracks, and traffic volume had a certain impact on fatigue cracks. Combined with the prediction results, it can be seen that when the traffic volume was small, the rising rate of the pavement fatigue crack curve was relatively slow, indicating that the fatigue crack development was relatively slow. However, whether the traffic volume was large or small, the final values of the two were similar, indicating that the size of traffic volume could only affect the development rate of fatigue cracks but could not change the final state of pavement fatigue cracks. This is because once the fatigue crack is generated, it will continue to develop under the action of alternating stresses, forming the crack propagation area and expanding continuously.

### Temperature crack

**Fig. 9:**
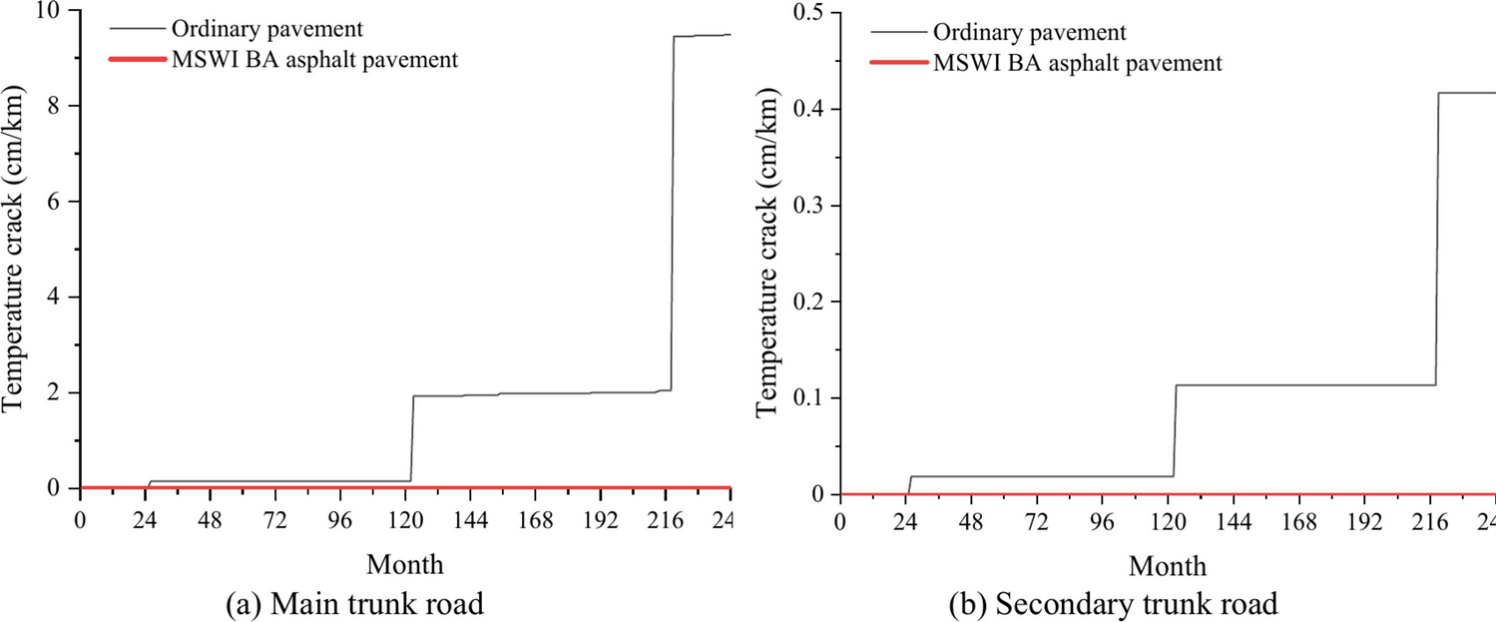
Prediction results of temperature crack.

The temperature cracks curves of the ordinary pavement and asphalt pavement containing MSWI BAA for the main and the secondary trunk roads with time can be seen in Fig. 9. The predicted maximum values of the temperature cracks of the four combinations were far less than the limit value of 189.39 cm/km. Especially, the prediction results of the asphalt pavement containing MSWI BAA were all 0. For the ordinary pavement, the trend of the temperature crack curves of the main and the secondary trunk roads showed a ladder shape. The maximum temperature crack of the main trunk road was 9.45 cm/km at 219 months, and the maximum temperature crack of the secondary trunk road was 0.42 cm/km at 219 months.

The temperature cracks of four kinds of pavement structure combination met the limit requirements, especially for asphalt pavement containing MSWI BAA, with a stronger ability to resist temperature. In addition, this pavement had an obvious inhibitory effect on the generation of temperature cracks and reduced the influence of pavement damage by temperature cracks.

Temperature cracks are cracks caused by low temperature or temperature cycle on the road surface, usually manifested as transverse cracks roughly perpendicular to the center line of the sidewalk. These cracks may be caused by HMA surface shrinkage or asphalt hardening caused by low temperature. Therefore, when the ambient temperature is constantly changing, the pavement is in the process of continuous shrinkage and expansion, and the influence of temperature on the pavement is in a process of continuous accumulation. When the accumulation reaches the critical value, a little influence is given, and the temperature crack is produced, thus showing the condition of temperature crack mutation. Then the influence of temperature will enter a new accumulation process, and this process is based on the process of the previous mutation, so when the new critical value is exceeded, the mutation will be more intense, showing that the temperature crack mutation is greater. Relevant experimental studies^40^ have shown that the interfacial adhesion between MSWI BAA and asphalt is better than that of natural aggregate, and the adhesion is stronger and less prone to mutation. It can be verified that the addition of MSWI BAA can improve the low temperature crack resistance of asphalt mixture.

### Pavement smoothness

**Fig. 10:**
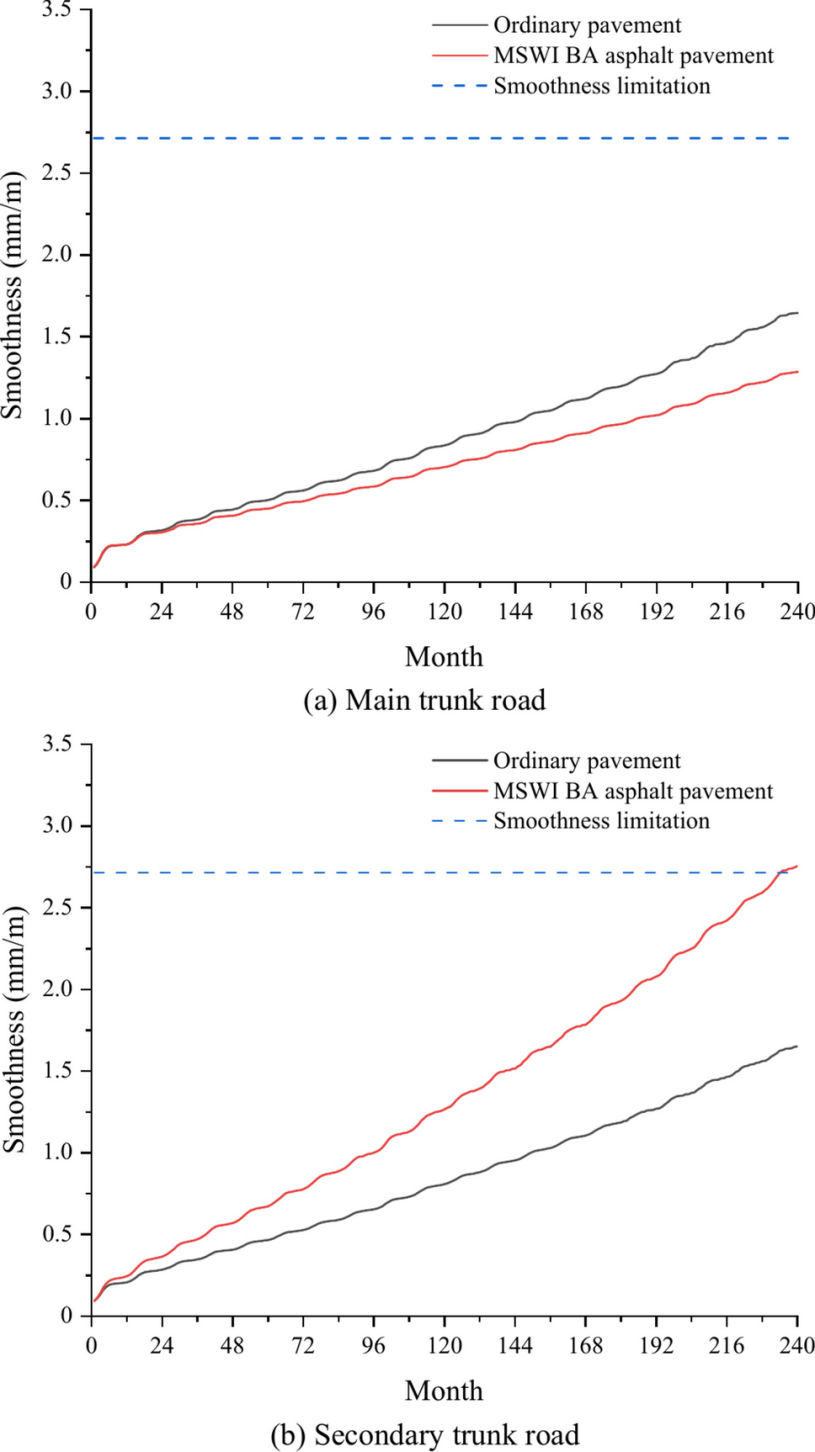
Prediction results of pavement smoothness.

Figure 10 demonstrates the smoothness curves of the ordinary pavement and asphalt pavement containing MSWI BAA for the main and secondary trunk roads with time. The smoothness of the four combinations of pavement increased gradually with time, and the growth rate was relatively stable, close to linear growth. For the main trunk road, the pavement smoothness of the ordinary road in the 240th month was 1.65 mm/m, and that of the asphalt pavement containing MSWI BAA in the 240th month was 1.29 mm/m. For the secondary trunk road, the pavement smoothness of the ordinary road in the 240th month was 2.71 5 mm/m, and that of the asphalt pavement containing MSWI BAA in the 240th month was 1.65 mm/m.

Compared with ordinary pavements, the smoothness of the main trunk road of asphalt pavement containing MSWI BAA was reduced from 1.65 to 1.29 mm/m, by 21.8%; the pavement smoothness of the secondary trunk road decreased from 2.71 to 1.65 mm/m, with a decrease of 39.1%. In addition, the smoothness of the secondary trunk road was higher than that of the main trunk road, the smoothness of the ordinary pavement increased from 1.65 to 2.71 mm/m, an increase of 65.2%, and the smoothness of the asphalt pavement containing MSWI BAA increased from 1.29 to 1.65 mm/m, an increase of 27.9%. It can be seen that the smoothness performance of asphalt pavement containing MSWI BAA was better, especially the smoothness of secondary trunk road was significantly improved. This is because the chemical composition of MSWI BA contained active S_i_O_2_, Al_2_O_3_, and C_a_O, reflecting a certain degree of water hardness and pozzolanic activity^41^. Therefore, a small amount of hydration products may be produced in the road use process, playing a certain anchoring role in reducing the generation of various pavement cracks. In addition, the smoothness of the secondary trunk road was higher than that of the main trunk road. The reason for this phenomenon is that there was no middle layer in the pavement structure of the secondary trunk road compared with the main trunk road, which decreased the pavement thickness and facilitated pavement damage. The smoothness value did not decrease but increased. Therefore, the different levels of pavement structure had a significant impact on pavement smoothness.

### Comprehensive analysis of road performance of BA asphalt pavement

In this chapter, the prediction results of rut, fatigue crack, temperature crack and pavement smoothness are briefly described and analyzed.

**Fig. 11:**
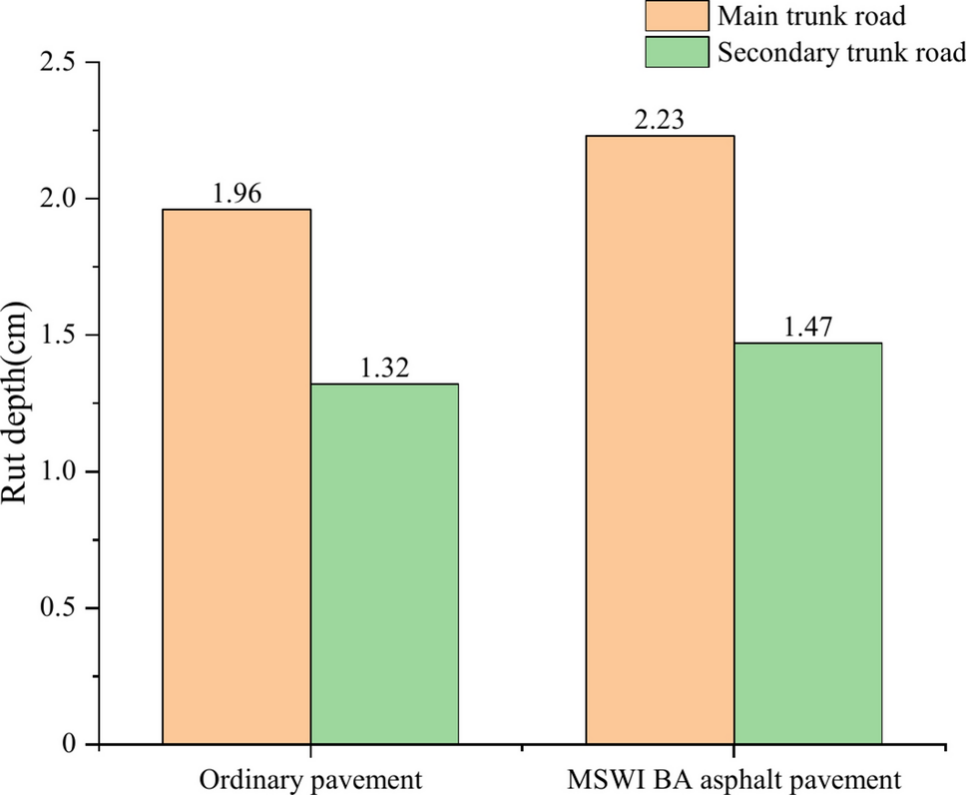
Rutting comparison chart of asphalt pavement under different traffic volume.

It can be seen from Fig. 11 that the rut depth of the main road is 32.7% deeper than that of the secondary road on the ordinary pavement, while the rut depth of the main road on the MSWI BA asphalt pavement is 34.1% deeper than that of the secondary road, which once again confirms that the incorporation of BA reduces the rutting resistance of asphalt mixtures and has a certain influence on the rut depth of the pavement.

**Fig. 12:**
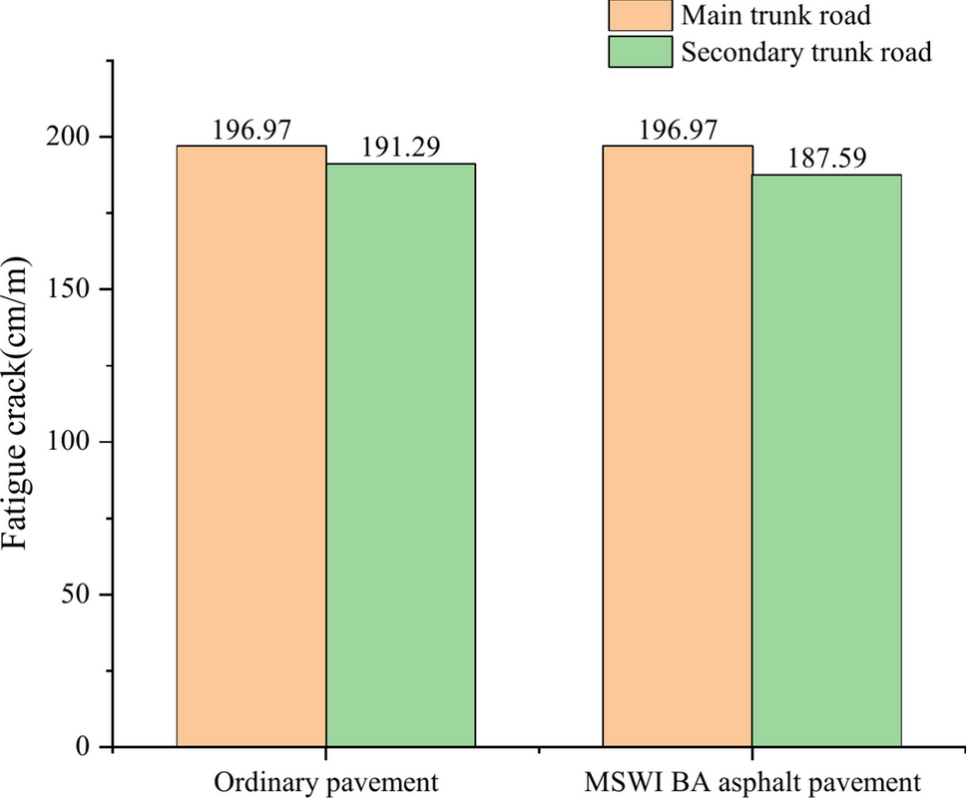
Comparison of fatigue cracks of asphalt pavement under different traffic volume.

It can be seen from Fig. 12 that the fatigue crack of the main road on the ordinary pavement is 2.9% deeper than that of the secondary road, while the fatigue crack of the main road on the MSWI BA asphalt pavement is 4.6% deeper than that of the secondary road, which once again confirms that the incorporation of BA has a certain impact on the fatigue crack of the pavement, but the impact is not significant.

**Fig. 13:**
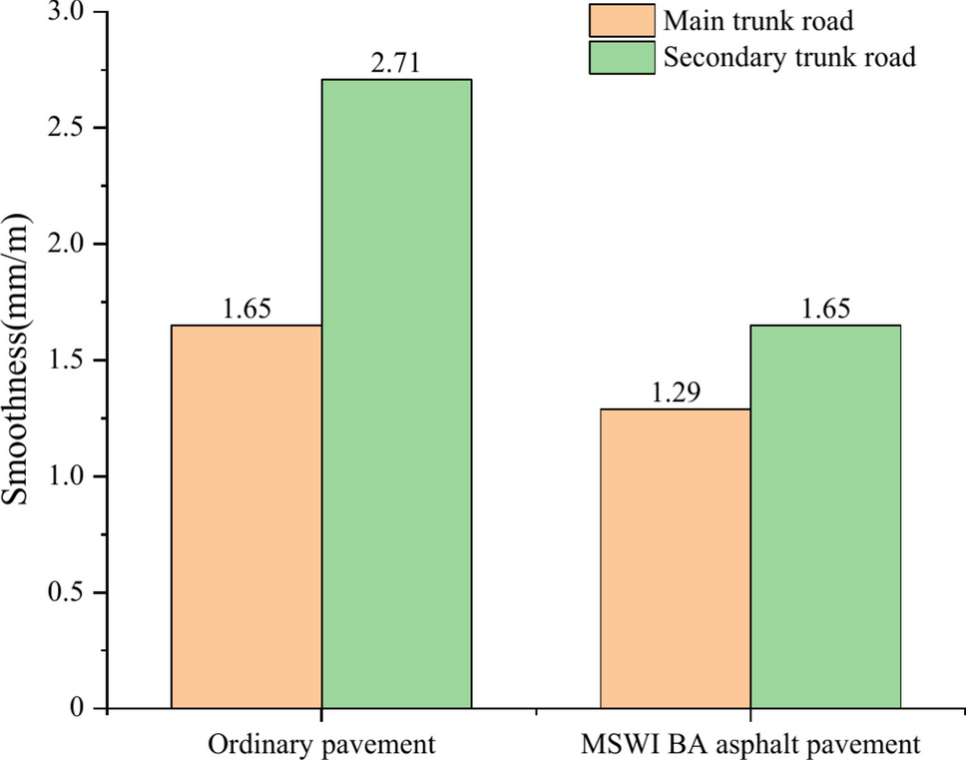
Comparison diagram of asphalt pavement smoothness under different traffic volume.

From Fig. 13, it can be seen that the pavement smoothness of the secondary trunk road on the ordinary pavement is 64.2% higher than that of the main trunk road, while the pavement smoothness of the secondary trunk road on the MSWI BA asphalt pavement is only 27.9% higher than that of the main trunk road, indicating that the pavement smoothness mixed with BA is better.

## Conclusion

In this paper, the Mechanistic-Empirical Pavement Design Guide (MEPDG) prediction model was used to evaluate and predict the performance of asphalt pavement containing MSWI BAA, such as rutting, fatigue crack, temperature crack, and pavement smoothness. Then, the results were compared with those of ordinary asphalt pavements. The main conclusions are as follows:

1. The research in this paper is a prediction result that comprehensively considers the factors of climate environment and traffic volume, which is more comprehensive and objective than the previous prediction results only by material model parameters.
2. The prediction results of rutting showed that the rutting resistance of asphalt pavement containing MSWI BA would be reduced, and the maximum rutting was increased by about 10% compared with the ordinary pavement. The effect of traffic volume on the rutting depth was pronounced. The asphalt pavement containing MSWI BAA was more suitable for the secondary trunk road with small traffic volume, and the increase in rut mainly came from the middle layer.
3. The prediction results of fatigue cracks showed that the MSWI BA had no obvious influence on the fatigue cracks of the pavement. In addition, the traffic volume influenced the fatigue cracks, but the traffic volume could only affect the development rate of the fatigue cracks, and did not change the cracking degree of the fatigue cracks in the final pavement.
4. The prediction results of temperature cracks indicated that after adding MSWI BA, the influence of temperature changes on the pavement was significantly reduced. This significantly reduced the possibility of temperature cracks and effectively avoided pavement damage caused by temperature cracks.
5. The prediction results of pavement smoothness demonstrated that incorporating MSWI BA significantly reduced the pavement smoothness index. At the same time, the different pavement structure levels also had a significant influence on the pavement smoothness.
6. In general, the MSWI BA will not reduce the long-term performance of asphalt pavement, and is suitable for replacing some natural aggregates for road construction, which is conducive to the reuse of solid waste and truly turning waste into treasure.

## Data Availability

All data generated or analysed during this study are included in this published article.

## References

[1] Joumblat, R., Kassem, H., Elkordi, A. & Khatib, J. Use of alternative recycled fillers in bituminous mixtures: a review[M]. *Advance Upcycling of By-Products in Binder and Binder-Based Materials (Woodhead Publishing Series in Civil and Structural Engineering)*10.1016/B978-0-323-90791-0.00007-X (2024).

[2] Monteiro, L. S. V. E., Bandarra, B. S., Quina, M. J. & Coelho, P. A. L. F. A multidisciplinary evaluation of mixtures of municipal solid waste incineration bottom ash and mine tailings for sustainable geotechnical solutions[J]. *Constr. Build. Mater.* **455**, 139139 (2024).

[3] Wu, B. B., Song, W. & Pu, Z. H. Analysis on technology and management status of comprehensive utilization projects of bottom ash from municipal waste incineration plant[J]. *Environ. Sanitation Eng.* **27**(03), 9–11 (2019).

[4] Zhong, Z. F. Research on comprehensive treatment and resource utilization of incinerator slag[J]. *Low Carbon World* **11**(11), 5–6 (2021).

[5] Gu, Y., Ren, X. Y., Xu, B. Y. & Sun, X. B. Analysis on resource utilization of waste incineration slag[J]. *Mod. Chem. Res.* **01**, 111–114 (2023).

[6] Maria, I. et al. Characterisation of bottom ash from municipal solid waste incineration in Catalonia[J]. *J. Chem. Technol. Biotechnol.* **77**(5), 576–583 (2002).

[7] Tiewtoy, S., Moocharoen, W. & Kuptasthien, N. User-centred machinery design for a small scale agricultural-based community using quality function deployment[J]. *Int. J. Sustain. Eng.* **17**(1), 1–14 (2023).

[8] Zhu, S. H. & Dan, Z. Research status and development of municipal solid waste incineration slag in China[J]. *Environ. Ecol.* **5**(02), 91–96 (2023).

[9] Becquart, F. et al. Monotonic aspects of the mechanical behavior of bottom ash from municipal solid waste incineration and its potential use for road construction[J]. *Waste Manag.* **29**(4), 1320–1329 (2009). 18977129 10.1016/j.wasman.2008.08.019

[10] Sun, W. Z. Long-term application evaluation od incineration slag aggregate in the road base course[J]. *Shanghai Highways* **1**, 6–9 (2015).

[11] Joumblat, R. et al. Characterisation of asphalt concrete mixes with municipal solid waste incineration fly ash used as fine aggregates substitution. *Int. J. Pavement Eng.*10.1080/10298436.2022.2099855 (2022).

[12] Yu, J. Experimental study on fatigue performance of domestic waste incinerator slag aggregate asphalt mixture[J]. *J. Highway Transp. Res. Devel.* **16**(10), 80–82 (2020).

[13] Liu, H. J., He, W. Z. & Zhang, W. L. Research on application of domestic waste incinerator slag fine aggregate in asphalt mixture[J]. *Traffic Transp.* **02**, 147–150 (2018).

[14] Zhang, C., Zhou, T., Liu, Y. & Xue, Y. Study on stabilization treatment of slag aggregate used in road[J]. *Northern Commun.* **01**, 53–56 (2022).

[15] Yang, C., Li, Q. & Lin, S. Study on mix proportion design and performance of slag asphalt mixture[J]. *Energy Conserv. Environ. Prot. Transp.* **18**(01), 80–83. 10.3969/j.issn.1673-6478.2022.01.018 (2022).

[16] Xu, L., Du, Y., Loprencipe, G. & Moretti, L. Rheological and fatigue characteristics of asphalt mastics and mixtures containing municipal solid waste incineration (MSWI) residues[J]. *Sustainability* **15**(10), 8356 (2023).

[17] Ding, Y. et al. Adhesion property of municipal solid waste incinerator bottom ash and limestone with asphalt based on surface energy theory[J]. *J. Adhes.* **10**, 923–946 (2024).

[18] Li, H. T., Guan, Y. S., Jia, Y. & Han, C. The study on long-term performance evolution of epoxy asphalt steel bridge deck pavement[J]. *J. Cn. Foreign Highway* **30**(06), 230–233 (2010).

[19] Zhu, L. & Li, Q. Long-term performance observation and analysis based on different base asphalt pavement[J]. *J. Cn. Foreign Highway* **36**(06), 60–63 (2016).

[20] Jiang, X. Road performance of reclaimed asphalt mixture mixed with bagasse fibers and rubber-modified asphalt[J]. *J. Cn. Foreign Highway* **44**(02), 130–137 (2024).

[21] Joumblat, R. et al. Performance evaluation of hot-mix asphalt with municipal solid waste incineration fly ash using the stress sweep rutting test[J]. *Innov. Infrastruct. Solut.* **8**, 261 (2023).

[22] Shi, D. S. et al. Analysis of microstructure and compressive strength of concrete with municipal solid waste incineration bottom ash as sand based on nuclear magnetic resonance technology[J]. *Bull. Chi. Ceram. Soc.* **42**(01), 248–257 (2023).

[23] Liu, D. & Li, L. H. Cementations properties of municipal solid waste incineration bottom ash aggregate[J]. *J. Tongji Univ. (Nat. Sci.)* **45**(3), 377–383 (2017).

[24] Sun, Yu., Li, L. H. & Huang, C. W. fundamental properties of asphalt mixture containing municipal solid waste incineration bottom ash[J]. *J. Build. Mater.* **23**(04), 978–983 (2020).

[25] Liu, D., Li, L. H. & Cui, H. J. Experimental study on influence of municipal solid waste incineration bottom ash aggregate on properties of asphalt mixture[J]. *J. Build. Mater.* **18**(2), 307–311 (2015).

[26] Liu, D., Li, L. H. & Yang, K. Durability of asphalt mixture containing municipal solid waste incineration bottom ash aggregate[J]. *J. Tongji Univ. (Nat. Sci.)* **44**(1), 100–106 (2016).

[27] Hu, M. J., Li, L. H., Wang, J. C. & Yang, K. Experimental study on pavement performance and leaching characteristic of asphalt mixture with bottom ash aggregate[J]. *J. Build. Mater.* **22**(03), 480–486 (2019).

[28] Sun, Y. *Interface characteristics of municipal solid waste incinerator bottom ash-asphalt and the influence on durability of asphalt mixture[D]* (Tongji Univ., 2018).

[29] Moins, B., Hernando, D., Seghers, D., Audenaert, A. & Van den bergh, W.,. Using mechanistic-empirical simulations and nonparametric testing to explore variability in the expected performance of reference asphalt pavement designs[J]. *Constr. Build. Mater.*10.1016/j.conbuildmat.2024.136374 (2024).

[30] Jablonski, B., Regehr, J., Rempel, G. GUIDE FOR MECHANISTIC-EMPIRICAL DESIGN OF NEW AND REHABILITATED PAVEMENT STRUCTURES[J]. *Final Report Part Design Analysis*, (2001).

[31] Kong, W., Huang, W., Guo, W. & Wei, Y. Modification of MEPDG rutting model based on RIOHTrack data. *Int. J. Pavement Eng.*10.1080/10298436.2023.2201500 (2023).

[32] Tilaka, W., Bambang, S. S., Sony, S. W. & Eri, S. H. Comparative analysis of overlay thickness using the asphalt institute’s and MEPDG with kenlayer[J]. *Int. J. GEOMATE* **26**(118), 57–64 (2024).

[33] Liu, W. & Lin, Z. Study on area correction factor of rutting prediction model for flexible base asphalt pavement based on MEPDG[J]. *J. Highway Transp. Res. Devel.* **34**(6), 67–70 (2017).

[34] Wang, H., Zhang, C., You, Z. & Chen, X. Calibration of rutting prediction model in MEPDG based on mathematical statistics method[J]. *J. Chang’an Univ.: Nat. Sci. Ed.* **33**(6), 15–18 (2013).

[35] Dinegdae, Y. H. & Birgisson, B. Effects of truck traffic on top-down fatigue cracking performance of flexible pavements using a new mechanics-based analysis framework[J]. *Rd. Mater. Pavement Des.* **19**(1), 182–200 (2016).

[36] Vamegh, M. & Ameri, M. Rutting performance of road pavement asphalt binders modified by polymers[J]. *Proc. Inst. Civ. Engr.-Constr. Mater.*10.1680/jcoma.17.00073 (2020).

[37] Gogoi, R. Prediction of longitudinal cracking of asphalt pavements[J]. *Int. J. Recent Technol. Eng. (IJRTE)*10.35940/ijrte.C3845.098319 (2019).

[38] Kırbaş, U. IRI sensitivity to the influence of surface distress on flexible pavements[J]. *Coatings*10.3390/coatings8080271 (2018).

[39] Ji, Y. & Xu, B. Application of domestic garbage incinerator residue in trial roads of pudong in shanghai and its post evaluation[J]. *Urban Rd. Br. Flood Control.* **04**, 186–190 (2019).

[40] Chen, F. et al. Research progress on test methods of asphalt mixture’s low - temperature anti - cracking performance[J]. *Mater. Rep.* **35**(S2), 127–137 (2021).

[41] Qin, L., Li, J., Ma, Y. & Yan, Q. Study on the influence of blast bottom ash as the aggregate on low - temperature performance of asphalt road[J]. *Western Cn. Commun. Sci. Technol.* **08**, 18–21 (2018).

